# Electroluminescence from *μ*LED without external charge injection

**DOI:** 10.1038/s41598-020-65092-z

**Published:** 2020-05-15

**Authors:** Kun Wang, Ye Liu, Chaoxing Wu, Dianlun Li, Shanhong Lv, Yongai Zhang, Xiongtu Zhou, Tailiang Guo

**Affiliations:** 1 College of Physics and Information Engineering, Institute of Optoelectronic Technology, Fuzhou University, Fuzhou, 350108 China; 2 Fujian Science & Technology Innovation Laboratory for Optoelectronic Information of China, Fuzhou, 350108 China

**Keywords:** Electronic devices, Inorganic LEDs

## Abstract

Stable electroluminescence from micro-pixelated light-emitting diode (*μ*LED) occurs when electrons and holes are continuously injected from external electrodes. Different from the general recognition, in this work, *μ*LED works in an operation mode, namely, non-electrical contact and non-carrier injection mode, and can be ‘wirelessly’ lit up without external charge injection, which is different from the general recognition. Inherent holes and electrons in *μ*LEDs can provide sufficient carriers for radiative recombination under alternating-current electric field. A possible model related to the diffusion of majority carrier and the drift of minority carrier in *μ*LED was proposed, which is further confirmed by the employment of a ‘carrier pump’. Finally, the intrinsic characteristics of the device-in-capacitor, namely, self-protection against electrical breakdown, were discussed. This work demonstrates a new device configuration and an alternative operating mode for *μ*LED and provides a research manner to obtain advanced *μ*LED-based technology.

## Introduction

GaN-based micro-pixelated light-emitting diodes (*μ*LEDs) have been emerging as promising photoelectronic platforms for advanced applications, including ultrahigh resolution displays, microdisplays, visible light communications, solid-state lighting, direct light writing and fluorescence-based micromanipulation^1–9^. The working mechanism of *μ*LED is clear. When a forward voltage is applied to the *μ*LED, the holes injected from the p region and the electrons injected from the n region recombine at the multi-quantum well (MQW) and generate spontaneous emission fluorescence. Continuous electrons and holes are injected from external electrodes under a forward bias, which leads to continuous electroluminescence. Therefore, an ohmic contact, which is essential for minimizing resistive losses and achieving high injection levels, must be made between the external electrode and the semiconductor^10^. Recently, a two-terminal light-emitting device based on single-layer semiconductors has been demonstrated, which is composed of a metal-semiconductor contact and a non-electrical contact^11^. Alternating electrons and holes are injected into the luminescence centre from the metal-semiconductor contact under alternating-current (AC) voltage. These results indicate that the combination of non-electrical contact and AC drive may provide an alternative operating mode for light emitting devices, especially GaN-based *μ*LEDs.

In this work, we propose a concept of lighting *μ*LEDs in a non-electrical contact and non-carrier injection (NEC&NCI) mode, in which carrier injection from external electrodes is completely prohibited. Inherent holes and electrons in the *μ*LEDs, rather than externally injected carriers, can provide sufficient carriers for radiative recombination in MQW. A possible operation model of the NEC&NCI mode related to the periodic oscillation of the carriers under AC electric field is proposed. This model is further experimentally confirmed by employing a ‘carrier pump’ for realizing a high light power density. Finally, we briefly discuss the mechanisms of the device’s intrinsic self-protection against electrical breakdown. This work demonstrates a device configuration and an alternative operating mode for *μ*LEDs and provides a research manner to obtain advanced *μ*LED-based technology.

## Results

**Figure 1:**
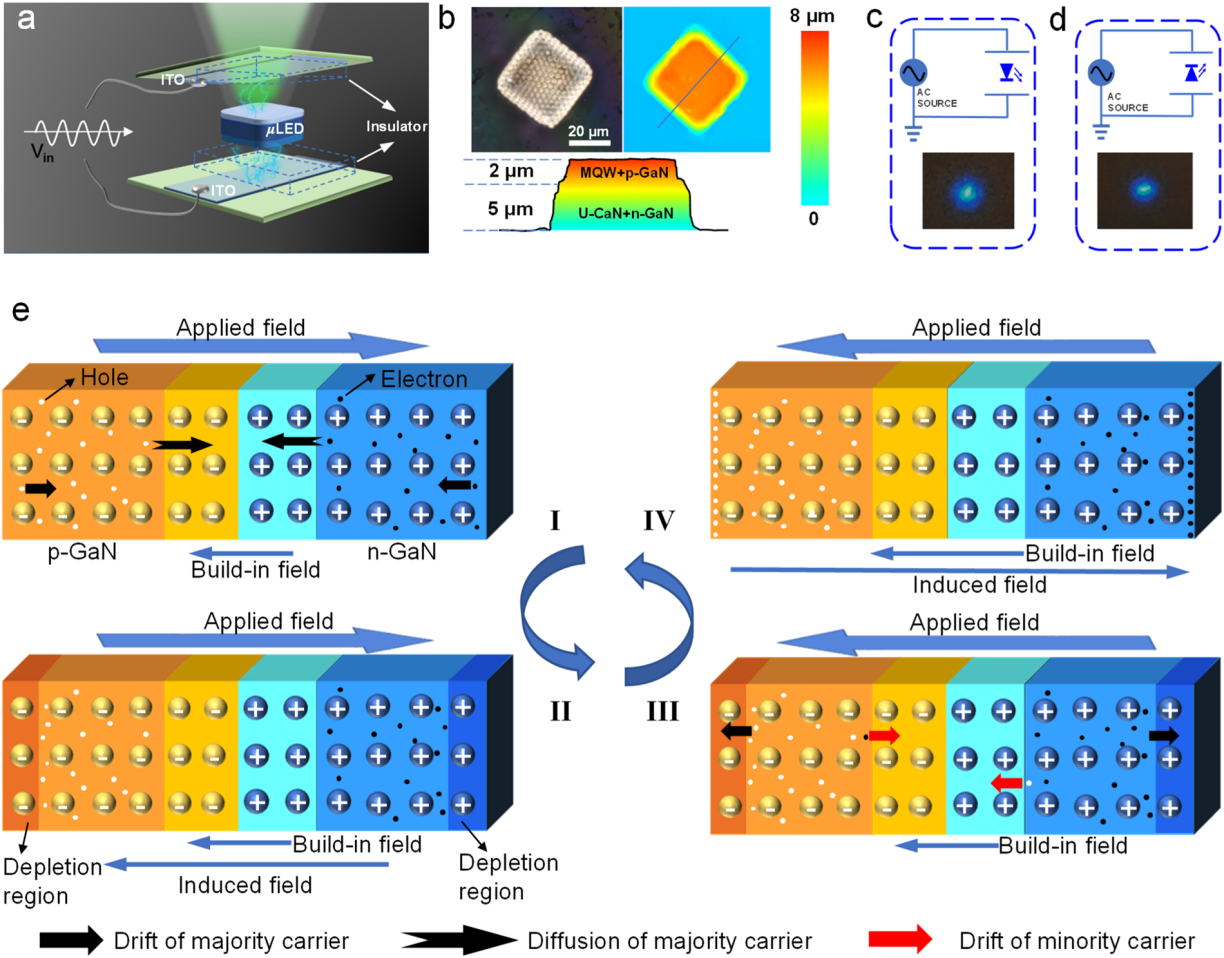
Device structure and operation mechanisms. (**a**) Schematic of the *μ*LED operated in NEC&NCI mode. (**b**) Optical microscopy image and 3D microscopy image of the *μ*LED. (**c,d**) Schematic and photographs of the *μ*LED in NEC&NCI mode, where the p-GaN of *μ*LED is facing up (**c**) and the n-GaN of *μ*LED is facing up (**d**). (**e**) Schematic showing the operation mechanisms of the *μ*LED in NEC&NCI mode. (e-I) Schematic showing the radiative recombination in the MQW region. (e-II) Schematic showing the formation of an induced electric field that shields the external field. (e-III) Schematic showing the movement of charge carriers when the applied bias polarity is opposite. (e-IV) Schematic showing the formation of an induced electric field.

A polyethylene terephthalate (PET, 125 μm thickness) layer s used as an insulating medium between the μLED and an external indium tin oxide (ITO) electrode, which can completely suppress external carrier injection. The stacked PET/*μ*LED/PET component is sandwiched between two ITO electrodes, as schematically illustrated in Fig. 1a (the fabrication process is shown in Fig. S1). An input AC voltage is applied to the ITO electrodes to light the *μ*LED. Our devices have the following advantages compared with conventional *μ*LED: (1) The *μ*LEDs used in this work have a simple structure of u-GaN/n-GaN/MQW/p-GaN (Fig. 1b). Conventional *μ*LED requires a transparent contact layer and an upper p-electrode to achieve efficient carrier injection (Fig. S2)^12,13^. However, the transparent contact layer, p-electrode, and n-electrode were eliminated in this work because external carrier injection was avoided. Their removal allows a more cost-effective mass production process for *μ*LED-based electronics. (2) Our device does not require charge injection from external materials; therefore, the elaborate design of energy band alignment for electron (hole) injection layer, transport layers, and emissive layers was eliminated^14,15^. (3) The ready-made *μ*LEDs have higher environmental stability and better performance compared with the light-emitting materials used in organic (quantum-dot) LEDs. (4) The output performances of our device are independent of the *μ*LED orientation as shown in Fig. 1c,d. Furthermore, we can observe the emitted light on both sides of the device because of the transparency of the 7 μm-thick *μ*LED (Fig. S3).

The possible operation processes of our device are schematically illustrated in Fig. 1e, We simplify the *μ*LED to a p–n junction and ignore the effect of MQW on the carrier motion in the following description. Electrons diffuse into the p-GaN region and holes diffuse into the n-GaN region under thermodynamic equilibrium to form a space charge region and a built-in field^16^. Electrons (majority carriers) in the n-GaN region move toward the MQW by diffusion under the forward bias. A similar process is performed on the p-side of the junction. Radiative recombination of electrons and holes occurs in the MQW region (Fig. 1e-I). However, light emission under the forward bias is transient, and only one flash can be observed when a constant bias is applied (Video S1). The reason is that the drift of majority carriers would cause the formation of a depletion region (or an inversion layer under a strong field) and generate an induced electric field to shield the external field (Fig. 1e-II). The shielding effect tends to limit the majority carrier’s diffusion and finally terminates the radiative recombination process.

The previously induced field would be removed due to the drift of majority carriers when the polarity of the applied bias was inverse, as schematically shown in Fig. 1e-III. In addition, due to the strong built-in field, a net current flowing in a direction from n-GaN to p-GaN is generated due to the strong built-in field. Simultaneously, the drift of the majority carriers in the bulk regions finally forms accumulation layers at the ends of p-GaN and n-GaN, respectively, and generated an induced electric field to shield the external field, as illustrated in Fig. 1e-IV. The device can maintain its periodic electroluminescence under AC voltage; therefore, we can infer that the total amount of charge transferred through the p–n junction under forward bias is the same as that under reverse bias. We propose a strategy based on this inference to enhance the electroluminescence, and this strategy will be discussed later.

**Figure 2:**
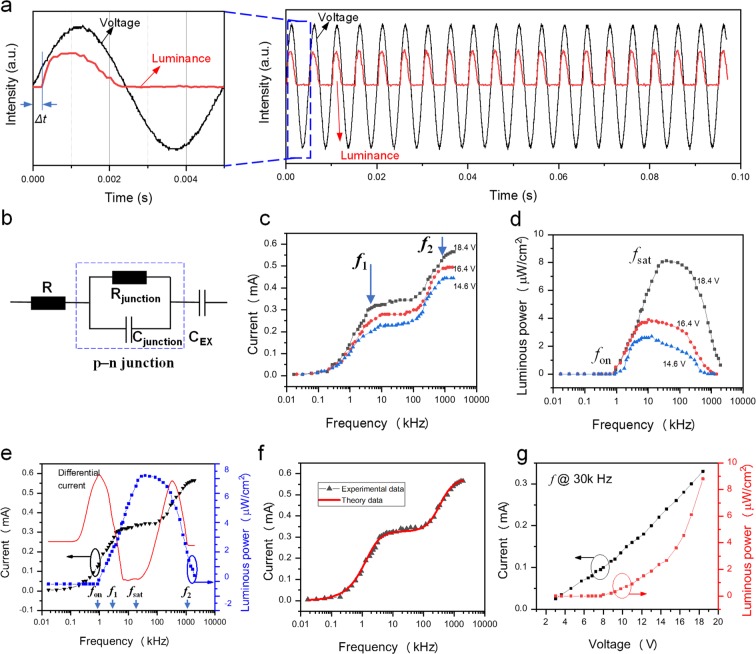
Electrical properties of the device. (**a**) Time-resolved electroluminescence. (**b**) Equivalent electric circuit diagram of the device. (**c**) Current–frequency relationship. (**d**) Luminescence power–frequency relationship. (**e**) Current, differential current, luminescence power and frequency relationship. (**f**) Experimental and theoretical current–frequency relationships. (**g**) Current–voltage and luminescence power–voltage relationships.

Figure 2a shows the time-resolved electroluminescence. Electroluminescence occurs only in the positive half of the period, which is consistent with our proposed model. In addition, luminescence intensity rapidly increases to its peak value and then gradually decrease in the rest time of the positive bias, because the gradual formation of an induced field under positive bias would shield the external field and gradually reduce the electroluminescence intensity.

From the perspective of circuit analysis, a *μ*LED is equivalent to resistor-capacitance (RC) parallel circuits, where R_LED_ is the resistance of bulk semiconductor and MQW, and C_LED_ is the capacitor related to p–n junction^16^. Furthermore, our device can be regarded as an RC circuit due to the presence of insulating PET layers, as shown in Fig. 2b. Therefore, the device is sensitive to the frequency of the applied AC voltages. The recorded current greatly depended on the operating frequency, and two corner frequencies (*f*_1_ and *f*_2_) are observed, as shown in Fig. 2c. The external capacitor (C_EX_) related to PET layers plays a major role in the impedance change at operating frequencies below *f*_1_, and the recorded current increase with frequency. When the frequency is slightly higher than *f*_1_, the capacitive reactance of C_EX_ is almost zero, and the current is independent of frequency. However, further increase in frequencies makes C_LED_ play a major role in impedance change. The capacitive reactance of C_LED_ decreases with increasing frequency when the operating frequency is slightly lower than *f*_2_, and the current increases with frequency. When the frequency is higher than *f*_2_, the capacitive reactance of C_LED_ is almost zero when the frequency was higher than *f*_2_, and the current approaches saturation again.

Electroluminescence intensity is also sensitive to operation frequency. Figure 2d presents the luminance–frequency characteristics of the device under different applied voltages. Electroluminescence is too weak to be observed in the low frequency range (<1k Hz). The electroluminescence intensity dramatically increases at a turn-on frequency of about 1k Hz (defined as *f*_on_) and continues to increase with frequency and finally reaches the maximum at the saturation frequency (*f*_sat_).

The corresponding differential current–frequency, current–frequency and electroluminescence–frequency characteristics are provided in Fig. 2e to show the relationship between the recorded current and the electroluminescence intensity clearly. For the differential current–frequency curve, the frequency (first peak) where the current changes rapidly is the same as *f*_on_. Thus, we can conclude that the increase in frequency (near *f*_on_) dramatically increases the operating current and causes more radiative recombination in the MQW region. According to circuit theory, the capacitive reactance of C_EX_ decreases as the frequency increases and thus increases the operating current and the voltage component applied to the p–n junction. Thus, electroluminescence intensity increases with frequency. However, when the frequency is further increased to a range between *f*_sat_ to *f*_2_, the C_LED_ plays the major role in the current change, and the electroluminescence intensity decreases with increasing frequency. From circuit theory, the decrease in C_LED_ capacitive reactance would decrease the current flowing through the R_LED_ and thus result in a decrease in the number of recombined carriers in the MQW region. Because the transit-time of the majority carriers from the bulk semiconductor to the MQW is constant, as the operating frequency increases, the number of majority carriers that can diffuse into the MQW during the positive half of the period decreases. In other words, the carrier transport cannot keep up with the changes in the applied field and will result in a decrease in electroluminescence intensity^17^. An extremeness case is that all majority carriers do not have enough time to diffuse to the MQW when the frequency increases to *f*_2_. As a result, no electroluminescence can be observed, as shown in Fig. 2e.

We calculate the current–frequency relationship of the *μ*LED operating in NEC&NCI mode by using phasor analysis. Note that phasor analysis does not apply to nonlinear circuit elements. However, the electrical properties of a *μ*LED operating in the NEC&NCI mode are different from that of a conventional *μ*LED under DC voltage. As will be discussed later, the total charge carriers transmitted through the p–n junction under forward bias should be the same as that under reversed bias. Thus, the rectification characteristics of the device are not obvious. Furthermore, the measured current curve has a sinusoidal shape (Figure S4). Therefore, we believe that phasor analysis has certain reference value for understanding the electrical performances of our device. As shown in Fig. 2f, the theoretical values are in good agreement with the experimental values, which clearly shows the effectiveness of our model (Note S1).

In addition, the current–voltage (*I-V*) relationship at a fixed frequency is demonstrated, as shown in Fig. 2g. Interestingly, the current is almost linearly related to the voltage in the measure range. This result is different from the *I-V* relationship of LED in normal operation mode. The possible reason is that the vector sum of resistance and capacitive reactance can be considered constant at a fixed frequency. The linear *I-V* curve can further prove the effectiveness of our RC equivalent mode. The wall-plug efficiency (*η*) of this device can be calculated as:

$${\eta }=\frac{{P}_{{\rm{LED}}}}{{P}_{{\rm{AC}}}-{P}_{{\rm{R}}}}$$

where *P*_LED_ is the output luminous power of LED, *P*_AC_ is the input power of the AC supply and P_R_ is the power consumption of a current-limiting resistance. As shown in Fig. 2g, the estimated *η* is 0.37% when the device reaches the maximum brightness at 30 kHz (**Note S2** and Figure S5).

**Figure 3:**
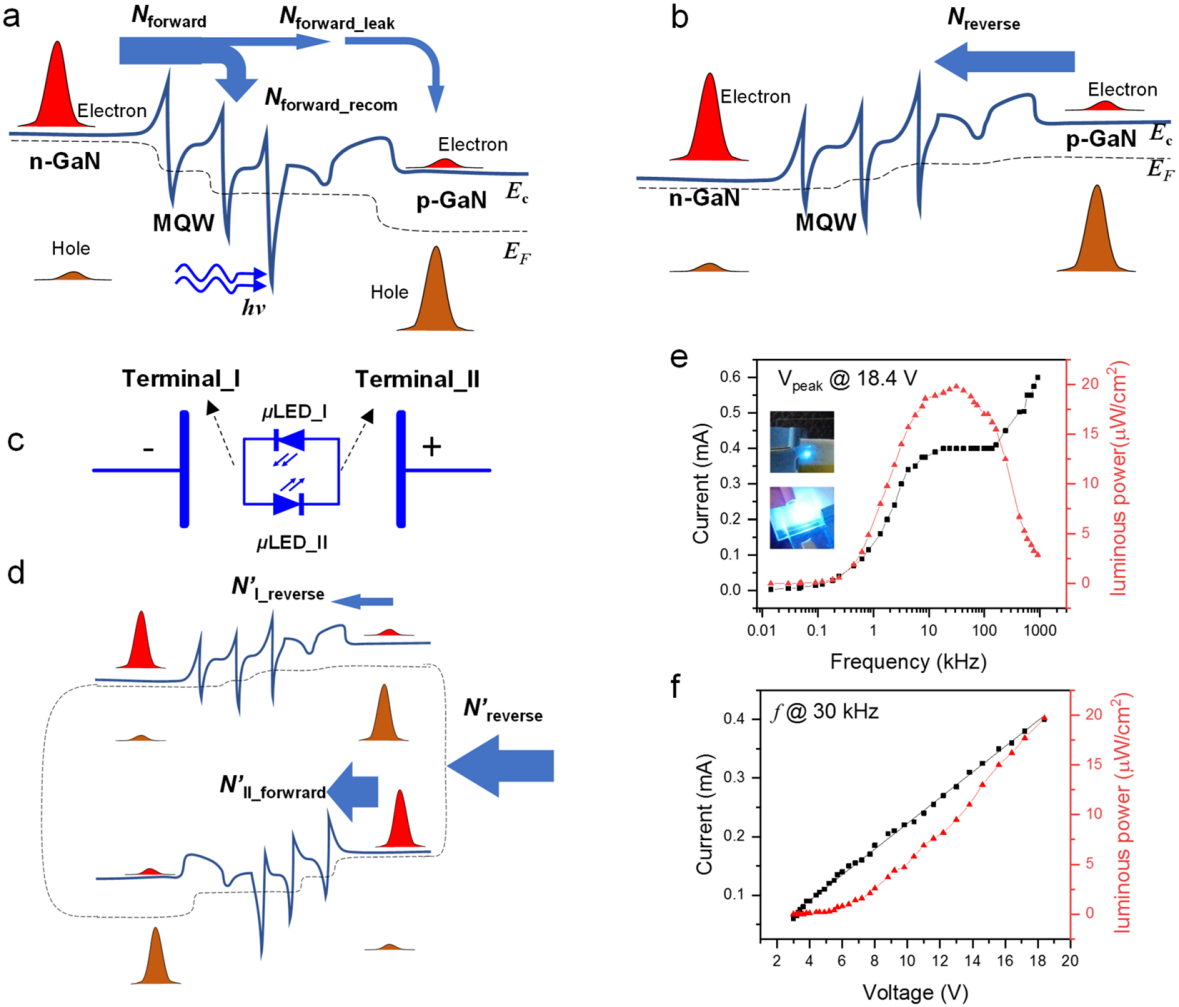
Strategy for performance improvements. (**a**) Schematic diagram showing the electron transfer under forward bias. (**b**) Schematic diagram showing the electron transfer under reverse bias. (**c**) Schematic diagram of the antiparallel *μ*LED pair. (**d**) Schematic diagram showing the electron transfer in the antiparallel *μ*LED pair under reverse bias. (**e**) Current–frequency and luminescence power–frequency relationships of the antiparallel *μ*LED pair. Insets are the photographs of a single *μ*LED (top) and an antiparallel *μ*LED pair (bottom). (**f**) Current–voltage and luminescence power–voltage relationships of the antiparallel *μ*LED pair.

It is well known that the electroluminescence intensity depends on the number of radiation-recombination carriers (Fig. 1e-I). Radiation-recombination carriers include the electrons diffused from n-GaN and the holes diffused from p-GaN^14^. Note that the diffusion of majority electrons and holes are similar; thus, we only consider the electrons diffused from n-GaN in the following discussion, as shown in Fig. 3. The net diffused electrons (*N*_forward_) in a forward-biased p–n junction that can be divided into two parts: (1) The electrons that recombine with holes (*N*_forward_recom_) that contribute to electroluminescence and (2) the leaked electrons (*N*_forward_leak_) that directly diffuse into the p-GaN region without recombination (Fig. 3a). This ratio can be considered an approximative constant because the ratio of *N*_forward_recom_/*N*_forward_ mainly depends on the *μ*LED structure. Thus, to enhance the electroluminescence, the *N*_forward_ should be increased.

The total carriers transmitted through the p–n junction under forward bias should be the same as that under reverse bias, because the device can maintain its periodic electroluminescence under AC voltage. Thus, the number of the net minority electrons (*N*_reverse_) in the reverse-biased p–n junction must be the same as the *N*_forward_. However, very few electrons drifted through the p–n junction under reverse bias (Fig. 3b). As a result, the *N*_forward_ that contributes to electroluminescence is clamped to the *N*_reverse._ In other word, we can infer that electroluminescence intensity is limited due to the low reversed current under reverse bias.

Based on the above inference, wWe propose a strategy to increase electroluminescence density by employing an antiparallel *μ*LED pair (*μ*LED_I and *μ*LED_II), as schematically presented in Fig. 3c. For the antiparallel *μ*LED pair, the p-GaN terminal of *μ*LED_I is electrically connected to the n-GaN terminal of *μ*LED_II, and the n-GaN terminal of *μ*LED_I is electrically connected to the p-GaN terminal of *μ*LED_II. We only consider the electroluminescence from *μ*LED_I in the following discussion.

The net electrons (*N’*_forward_) driven from terminal_I to terminal_II include two parts when the antiparallel *μ*LED pair is forward-biased (defined as *μ*LED_I is under forward bias): (1) Electrons diffuse through the forward-biased *μ*LED_I (*N’*_I_forward_), which predominate the *N’*_forward_; (2) Electrons drift through the reverse-biased *μ*LED_II (*N’*_II_reverse_). In this case, the electroluminescence from *μ*LED_I can be observed. Analogously, when the antiparallel *μ*LED pair is under reverse bias, electrons (*N’*_reverse_) will be driven from terminal_II to terminal_I. As shown in Fig. 3d, the *N’*_reverse_ also includes two parts: (1) Electrons diffuse through the forward-biased *μ*LED_II (*N’*_II_forward_), which makes the most contribution to *N’*_reverse_; (2) Electrons drift through the reverse-biased *μ*LED_I (*N’*_I_reverse_). Obviously, the *N’*_reverse_ is the same as the *N’*_forward_, and *N’*_forward_ and *N’*_reverse_ are much larger than the *N*_reverse_ of the device based on single *μ*LED. Therefore, a large number of electrons can be driven from the p-GaN terminal of μLED_I to the n-GaN terminal through *μ*LED_II even under reverse bias. In other words, *μ*LED_II acts as a highly efficient ‘carrier pump’ for the reverse-biased *μ*LED_I. A strong electroluminescence can be observed in *μ*LED_I under forward bias. The electroluminescence density and operating current of the antiparallel μLED pair-based device are obviously increased compared with the device based on a single μLED, as shown in Fig. 3e,f.

**Figure 4:**
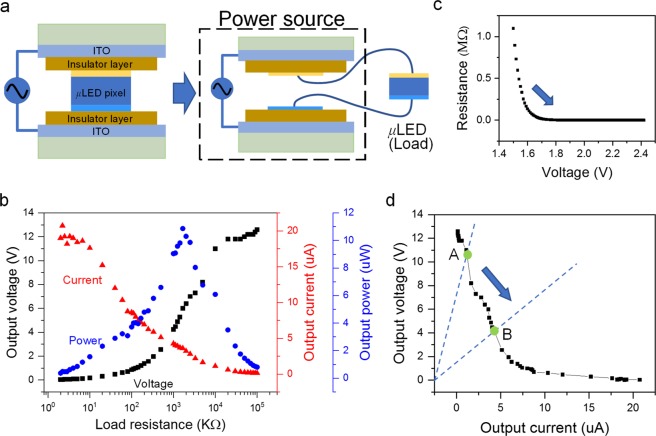
Self-protection against electrical breakdown. (**a**) Schematic of the DIC. (**b**) Resistance–voltage relationship of LED. (**c**) Output voltage–load resistance and output current–load resistance relationships of the DIC. (**d**) Operation voltage–current relationship of the LED operating in DIC mode.

The proposed μLED operating in the NEC&NCI mode can be equivalent to an electronic system of a *μ*LED embedded in a capacitor, namely, device-in-capacitor (DIC). In this DIC, two parallel ITO electrodes and insulator layers constitute a capacitor, and a *μ*LED is inserted into the insulator layers. Therefore, the capacitor connected to external AC power can be considered an equivalent power source for the embedded *μ*LED, as schematically presented in Fig. 4a. Notably, no carrier is injected from the equivalent power source to the embedded *μ*LED, and the equivalent power source exhibits different output characteristics. As shown in Fig. 4b, the output current, voltage and power are highly dependent on load resistance. In this measurement, the external AC power (*f* = 30 kHz, *V*_pp_ = 70 V) is applied, and a variable resistor is connected as the external load. Output peak current dramatically decreases as the load resistance increases and the output peak voltage increases with the load resistance. These events are different from those in a normal power source. Consequently, the maximum output power can be obtained at a specific load resistance. Output power as low as 11 μW can be observed even though a high voltage (35 V) is applied.

Interestingly, a self-protection function naturally exists in the DIC to prevent electrical breakdown due to the low output power. The resistance of nonlinear electronic element, such LED, depends on the applied voltage, as shown in Fig. 4c. The resistance of the LED will decrease when the LED is operating under a high positive bias (point A in Fig. 4d) and therefore reduces operation bias to avoid overcurrent (point B in Fig. 4d). The resistance dramatically decreases when the LED is operated under high negative bias close to the breakdown voltage threshold. As a result, the negative bias applied to the LED would also decreases to avoid breakdown. This reason explains why the *μ*LEDs in our device can operate normally under such high voltage.

## Discussion

One of the disadvantages of our device is the high operating voltage and AC frequency. Since the *μ*LED employed in this work is used to fabricate conventional μLED display, it may be not suitable for working in the NEC & NCI mode. The operating voltage and frequency can be reduced by optimizing the structure of the *μ*LED, including the thickness of the p-GaN and n-GaN and the impurity concentration.

Because the electrical contact between the external electrode and the *μ*LED is avoided, the proposed NEC & NCI mode operation mode may be applied to a conventional *μ*LED display. As well known, a large-scale precise electrical bonding technology, which might reduce the rate of working pixels and increase manufacture cost^1,18–20^, must be used for the hybrid integration of *μ*LED arrays with separate driving circuits. The inevitable contact resistance would increase the threshold voltage and heat generation and decrease the device’s operating lifetime^21,22^. No electrical contact is required between external electrode and the *μ*LED in our device. Thus, the current complicated microelectronic fabrication process for precise electrical bonding in *μ*LED display might be simplified or avoided when this technology is further developed in the future. Furthermore, the ‘wireless’ drive technology might promote the applications of *μ*LED, such as in the biomedical field.

In summary, periodic electroluminescence from *μ*LEDs operating in NEC&NCI mode is successfully demonstrated. Electroluminescence without external carrier injection are realised by applying an AC electric field, which can generate the periodic oscillation of inherent carriers in *μ*LED: The forward field causes the diffusion of majority carrier and subsequent radiative recombination in the MQW; the reverse field drifts the carriers to their original state to prepare for the next electroluminescent process. The performances of the *μ*LEDs operating in the NEC&NCI mode are highly sensitive to the applied AC frequency. Maximum electroluminescent intensity can be obtained at approximately 30 kHz. Furthermore, an antiparallel *μ*LED acting as ‘carrier pump’ is employed to increase the luminescence intensity, which experimentally confirms our proposed theoretical model. Finally, we demonstrate that the load-depended output of DIC leads to the device’s intrinsic self-protection against electrical breakdown. Our work presents an operating mode for *μ*LED without external carrier injection and presents a device configuration without electrical contact, which offers a new research manner to obtain advanced *μ*LED-based technology.

### Experimental details

#### Device Fabrication

*μ*LEDs (40 μm × 40 μm) composed of p-GaN, InGaN/GaN MQW and n-GaN were provided by Xiamen Changelight Co., Ltd. A laser lift-off process was performed to separate the 7 μm-thick *μ*LED layer from the sapphire substrate. A PET layer (125-μm thick) was attached on ITO glass to suppress the injection of external carriers completely from ITO. The *μ*LED was first transferred onto the surface of the PET-coated ITO glass. Then, another PET-coated ITO glass was vertically stacked on the former one (Figure S1). Thus, the *μ*LED was positioned between two vertically stacked PET layers.

For the antiparallel *μ*LED pair, a patterned ITO film (0.1 mm × 5 mm) was pre-formed on a PET layer, and the *μ*LEDs were transferred to the ITO surface. The p-GaN terminal of *μ*LED_I was in contact with the ITO, and the n-GaN terminal of the *μ*LED_II was in contact with the ITO. Another patterned ITO/PET layer was deposited on the *μ*LED pair. Finally, the as-assembled device was sandwiched between the ITO electrodes.

For the fabrication of the DIC, a PET layer (5 mm × 5 mm) was first deposited on ITO glass. Then, a copper layer was deposited on the PET surface. Two layers of ITO glass/PET/Cu were stacked opposite each other, and a 125 μm-thick PET film was inserted between the two copper layers. AC voltage was applied to the ITO electrodes, and the output signals were extracted from the two copper films.

#### Characterisation

AC sinusoidal waveform voltages were generated from a waveform generator (RIGOL, DG4162) and an amplifier (Agitek, ATA-122D). AC currents were measured by using an oscilloscope (RIGOL, DS1102E), and luminance was measured through a silicon photodiode (EVERFINE, FLA2000). Micrograph images were obtained via a 3D measuring laser microscope (Olympus, OLS4100). All measurements were performed in air at room temperature.

## Supplementary information

Supplementary Video S1.

Supplementary Information.
