# Supplementary material for: Electroluminescence from μLED without external charge injection
Source: Sci Rep. 2020 May 15;10:8059. doi: 10.1038/s41598-020-65092-z (PMC7229229; doi:10.1038/s41598-020-65092-z)
Supplement: Supplementary file 2 — Supplementary Information. [file 41598_2020_65092_MOESM2_ESM.pdf]

Electroluminescence from μLED without external charge injection

Kun Wang[1ǂ], Ye Liu[1ǂ], Chaoxing Wu[1,2*], Dianlun Li[1], Shanhong Lv[1], Yongai Zhang[1,2], Xiongtu Zhou[1,2*], Tailiang Guo[1,2*]

[1] College of Physics and Information Engineering, Institute of Optoelectronic Technology, Fuzhou University, Fuzhou, 350108, China

[2] Fujian Science & Technology Innovation Laboratory for Optoelectronic Information of China, Fuzhou, 350108, China

[ǂ] Both authors contributed equally to this work

[*] Corresponding author's e-mail: nnnwcx@foxmail.com (C. Wu), xtzhou@fzu.edu.cn (X. Zhou), gtl_fzu@hotmail.com (T. Guo)

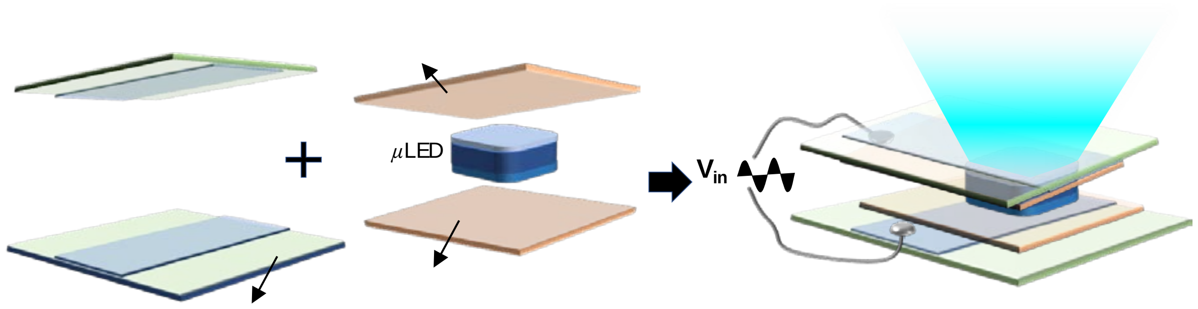

Figure S1. Fabrication process of μLED operated in NEC&NCI mode. A PET layer used as the insulating medium is deposited on the ITO glass to completely suppress the injection of charge carriers from external electrodes. The μLED is then transferred to a position between two vertically stacked PET layers, which is sandwiched between two ITO electrodes. AC voltage is applied to light up the μLED.

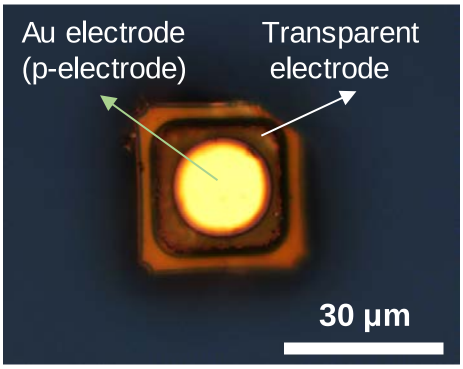

Figure S2. Optical microscopy image of a *μ*LED with electrode. A transparent contact layer and an upper p-electrode deposited on p-GaN are necessary for effective electrical contact between the *μ*LED and an external electrode.

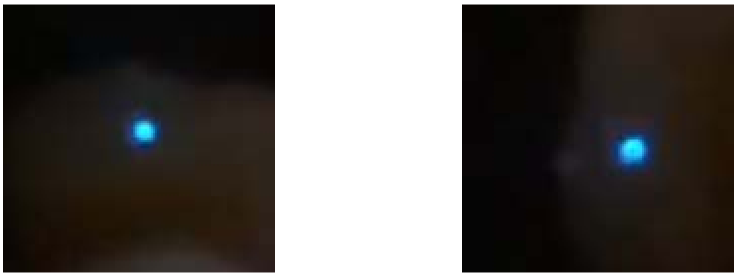

From the front side From the back side

Figure S3. Light can be emitted from both sides of a μLED operating in the NEC & NCI mode.

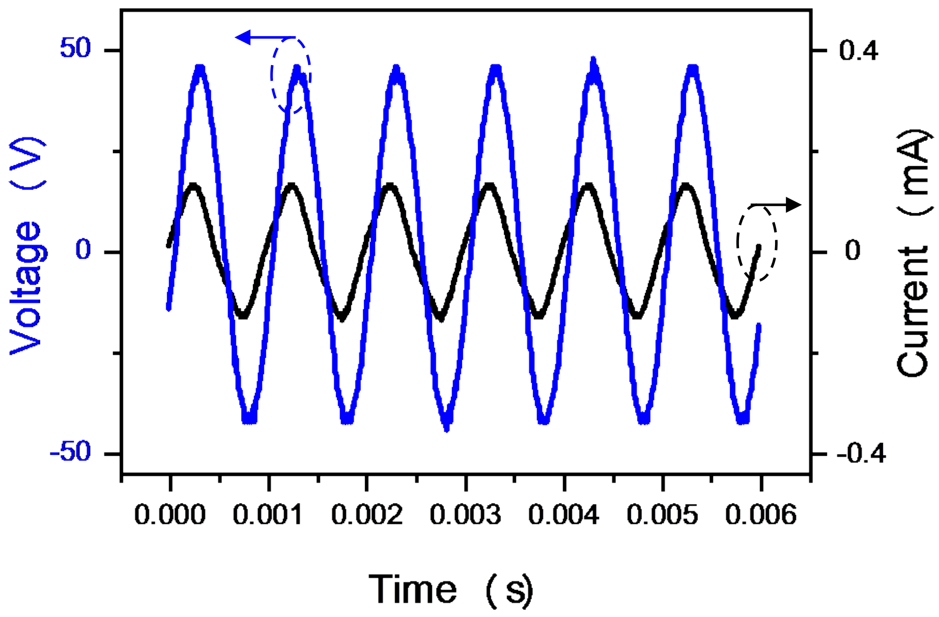

Figure S4. Applied voltage and measured current curves of the μLED operating in NEC & NCI mode.

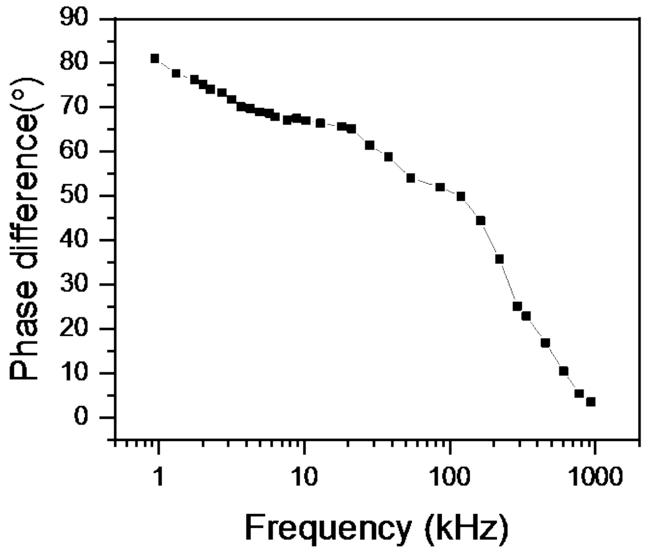

Figure S5. Phase difference between the measure AC current and the input AC voltage.

Note S1. The μLED in NEC&NCI mode can be considered as a RC circuit. A μLED can be equivalent to a RC parallel circuits, where R_{LED} is the resistance of bulk semiconductor and the MQW, and the C_{LED} is a capacitor related to the pn-junction. The external capacitor (C_{EX}) is related to the PET layers. The current can be given by:

$$\mathrm{I} = \frac{U}{Z}$$

$$\mathrm{Z} = \mathrm{R} + \frac{1}{2\pi f \mathrm{C_{EX}}\, \mathrm{j}} + \frac{1}{\frac{1}{\mathrm{R_{LED}}} + 2\pi f \mathrm{C_{LED}}\, \mathrm{j}}$$

Where f is the frequency of the applied AC voltage, and j is the imaginary unit. The current-frequency relationship can be calculated by using the above equation.

Note S2. The wall-plug efficiency (η) of this device can be estimated as:

$$\eta = \frac{P_{\mathrm{LED}}}{P_{\mathrm{AC}} - P_{\mathrm{R}}} = \frac{P_{irradiance} \times \mathrm{S}}{V \cdot I \cdot \cos(\alpha)/2 - I^2 R/2}$$

where P_{LED} is the output luminous power of LED, P_{AC} is the input power of the AC supply, P_{R} is the power consumption of a current-limiting resistance (R, 20 kΩ), and $P_{\mathrm{irradiance}}$ is the measured irradiance and S is (~0.8 cm^2). Because the sensitive area of the irradiance meter probe is much larger than the area of the μLED and the probe is tightly close to the μLED, the P_{LED} is estimated as $P_{\mathrm{irradiance}} \times \mathrm{S}$. V and I are the peak value of the input voltage and the measured current, respectively. α is the phase difference between the measured current and the input voltage (Figure S5). As shown in Figure 2g, when the device reaches the maximum brightness at 30 kHz, the $P_{\mathrm{irradiance}}$, V, I, α are 8.9 μW/cm^2, 18.4 V, 0.33 mA, and 60, respectively. Thus, the calculated η is 0.37 %.
